# The Stalk and 1B Domains Are Required for Porcine Deltacoronavirus Helicase NSP13 to Separate the Double-Stranded Nucleic Acids, and the Deletion of the ZBD Impairs This Activity

**DOI:** 10.3390/ani15060865

**Published:** 2025-03-18

**Authors:** Chengcheng Wu, Lihan Tao, Quanyong Zhou, Fanfan Zhang, Yanbing Zeng

**Affiliations:** 1 Institute of Animal Husbandry and Veterinary Medicine, Jiangxi Academy of Agricultural Sciences, Nanchang 330200, China; 2 College of Animal Science and Technology, Jiangxi Agricultural University, Nanchang 330045, China

**Keywords:** porcine deltacoronavirus, helicase, domains, unwinding activity, ATPase activity

## Abstract

The nonstructural protein 13 (NSP13) of PDCoV is a highly conservative helicase and plays key roles in viral replication. NSP13 contains a zinc-binding domain (ZBD), a helical Stalk domain, a beta-barrel 1B domain, and a core helicase domain. However, the specific functions of these domains of PDCoV NSP13 remain largely unknown. Here, we expressed and purified the wild-type NSP13^WT^ and various mutants with domain deletions, and the activities of these proteins were analyzed using multiple methods. We found that NSP13^ΔZBD^ possessed the abilities to hydrolyze ATP and unwind double-stranded nucleic acids, but the unwinding efficiency was lower than that of NSP13^WT^. In contrast, NSP13^ΔZBD-Stalk^, NSP13^Δ1B^, and NSP13^ΔZBD-Stalk-1B^ all lost their unwinding activity, but not their ATPase activity. These results revealed that the deletion of the ZBD impaired the unwinding activity of PDCoV helicase NSP13, and the Stalk and 1B domains were critical for NSP13 to separate the duplexes. The identification of the roles of each domain in this study was helpful to gain an in-depth understanding of the overall functions of helicase NSP13, providing a theoretical basis for the development of antiviral drugs targeting helicase.

## Simple Summary

The helicase NSP13, encoded by porcine deltacoronavirus (PDCoV), regulates viral replication and is considered to bean ideal target for antiviral drugs. In our work, the wild-type NSP13^WT^ and various deletion mutants were generated through expression and purification, and we tested the activities of these proteins using multiple methods. The results revealed that both the Stalk and 1B domains were necessary for NSP13^WT^ to separate the double-stranded nucleic acids, and the deletion of the ZBD impaired this unwinding activity. Our study is the first to explore the role of each domain in the activities of PDCoV NSP13, providing mechanistic insights into helicase-regulated viral replication and laying the foundation for the development of antiviral drugs.

## 1. Introduction

Porcine deltacoronavirus (PDCoV) is an enteropathogenic coronavirus that is capable of triggering diarrheal deaths in nursing piglets, causing significant economic losses to the global swine industry [1,2,3]. PDCoV also has the potential for cross-species transmission and the risk of transmission to humans, thus causing severe threats to the health of humans and animals [4,5,6]. The PDCoV genome is a linear single-strandedpositive-stranded RNA with a length of approximately 25.4 kb [7]. Upon the infection of its target cells, the genome of PDCoV is translated into two large replicative polyproteins that are subsequently processed into fifteen mature nonstructural proteins (NSP2-NSP16) via the papain-like protease (PLpro) and 3-chymotrypsin-like protease (3CLpro) [8]. The NSP13 of coronaviruses is a highly conservative helicase [9]. Helicases are known as motor proteins that translocate along the nucleic acid backbone and break the hydrogen bonds of base-paired nucleic acids by using the energy from nucleotide trisphosphate (NTP) hydrolysis; many aspects of nucleic acid metabolism require helicase-mediated strand separation. Helicases are divided into six superfamilies (SF1–SF6), and NSP13 belongs to the Upstream frameshift protein 1 (Upf1)-like subfamily of SF1 [10,11].

In a previous study, NSP13 was reported to unwind viral dsRNA intermediates to provide a single-stranded template for viral RNA amplification [12]. Severe acute respiratory syndrome coronavirus (SARS-CoV) helicase NSP13 separated dsRNA or dsDNA with a 5′ to 3′ polarity using the energy of NTP hydrolysis; the unwinding rate was ~280 base-pairs per second [13]. The helicase NSP13 of Middle East respiratory syndrome coronavirus (MERS-CoV) unwound DNA and RNA in a similar manner as SARS-CoV helicase, preferring to unwind the duplex substrates with longer 5′-overhangs; it exhibited significant differences in helicase activity in solutions containing different divalent metal ions [14]. Severe acute respiratory syndrome coronavirus 2 (SARS-CoV-2) helicase NSP13 was also able to use the energy derived from NTP hydrolysis for the translocation and unwinding of dsDNA and dsRNA in a 5′-to-3′ direction [15]. SARS-CoV-2 NSP13 preferentially interacted with ssDNA over ssRNA in order to unwind a partial duplex substrate; oligomerization was important for its optimal activity [16]. Porcine epidemic diarrhea virus (PEDV) helicase NSP13 hydrolyzed NTP and unwound dsRNA and dsDNA in a unidirectional fashion, and the unwinding activity of PEDV NSP13 was also affected by the divalent metal ion species [17].

NSP13 is currently considered as a potential target for antiviral drug development due to its critical role in viral genome replication [18,19,20]. For instance, the natural compound ZINC12899676 weakened the NTPase activity of PEDV NSP13 by targeting its active pocket, significantly inhibiting PEDV replication [21]. Ranitidine bismuth citrate inhibited the SARS-CoV-2 NSP13 activities in a dose-dependent manner, exhibiting low cytotoxicity and suppressing SARS-CoV-2 replication, leading to decreased viral loads in both the upper and lower respiratory tracts [22,23]. NSP13 consists of an amino terminal zinc-binding domain (ZBD), a helical Stalk domain, a beta-barrel 1B domain, and a core helicase domain, which contains Recombination-like domain 1A (Rec1A) and Recombination-like domain 2A (Rec2A) at the carbon terminus [24]. The core helicase domain comprises at least seven conserved amino acid motifs and includes the residues responsible for nucleotide binding and hydrolysis [25]. However, there is still a lack of research on the domain function of PDCoV NSP13.

Here, we focus on the ATPase and unwinding activities of PDCoV helicase NSP13 and its mutants; non-denaturing polyacrylamide gel electrophoresis (PAGE), the adenosine triphosphatase (ATPase) assay, and the fluorescence resonance energy transfer (FRET)-based unwinding assay are carried out in vitro. These studies will collectively reveal the functional roles of various domains in the activities of PDCoV helicase NSP13, providing a mechanistic foundation for the development of antiviral drugs targeting these domains.

## 2. Materials and Methods

### 2.1. Construction of the Recombinant Plasmids

The wild-type NSP13^WT^ sequences of PDCoV were downloaded from GenBank (accession no. MH025764.1) and synthesized by a biotech company (General Biol, Chuzhou, China). Thereafter, the DNA fragment was oriented and cloned into the vector pET-28a to construct the prokaryotic expression plasmid pET-28a-NSP13^WT^. The DNA sequences of various domain deletion mutants were also inserted into pET-28a, and the recombinant plasmids pET-28a-NSP13^ΔZBD^, pET-28a-NSP13^ΔZBD-Stalk^, pET-28a-NSP13^ΔZBD-Stalk-1B^, and pET-28a-NSP13^Δ1B^ were created. All recombinant plasmids were verified using DNA sequencing.

### 2.2. Expression of PDCoV Helicase NSP13^WT^ and Deletion Mutants

The recombinant plasmid pET-28a-NSP13^WT^ was transformed into the *E. coli* BL21(DE3) cells (Tsingke Biotech, Beijing, China) to express the enzymatically active NSP13^WT^. Monoclonal colonies were inoculated into 5 mL Luria–Bertani (LB) liquid medium containing kanamycin (Aladdin Biochemical Technology Co., Ltd., Shanghai, China) and were grown at 37 °C overnight. The cultures were grown in LB liquid medium at a ratio of 1:100 for expansion and were induced with 0.1 mM isopropyl-β-D-thiogalactopyranoside (IPTG) (Macklin Biochemical Co., Ltd., Shanghai, China) after the optical density at 600 nm (OD600) reached 0.6-0.8. Thereafter, the induced cells were grown at 16 °C in a constant temperature shaker (Zhichu Instrument Co., Ltd., Shanghai, China) at 90 rpm/min for 20 h. The expression processes of NSP13^ΔZBD^, NSP13^ΔZBD-Stalk-1B^, and NSP13^Δ1B^ were similar to that of NSP13^WT^; however, NSP13^ΔZBD-Stalk^ was induced in a constant temperature shaker at 60 rpm/min.

### 2.3. Purification of Recombinant Proteins

Cells were harvested via centrifugation at 5000 rpm/min and were resuspend in ice-cold phosphate-buffer saline (PBS) solution (10 mM NaH_2_PO_4_, 140 mM NaCl, and 3 mM KCl; pH = 7.8) three times, before the supernatant was discarded. The cells were resuspended again with the buffer (25 mM HEPES-KOH, 1 M NaCl, 10 mM Na_2_S_2_O_5_, 5 mM MgCl_2_, 1.6 mM imidazole, 7 mM β-mercaptoethanol, 1 mM phenylmethylsulfonyl fluoride, and 30% glycerol; pH = 7.8) and were crushed using an ultrasonic crusher (Scientz, Ningbo, China). The mixture solution was centrifuged at 12,000 rpm/min for 40 min, the supernatant was collected and filtered through a 0.25 μm disposable syringe filter (Millipore, Burlington, MA, USA), and this was then run through a Ni-affinity column (Genscript Biotech Corporation Co., Ltd., Nanjing, China). The proteins were eluted with a linear gradient concentration of imidazole from 10 to 300 mM. The purified products were concentrated using the ultrafiltration centrifugal tubes (Millipore, Burlington, MA, USA) and were stored in the buffer (25 mM HEPES-KOH, 0.1 M NaCl, 1mM dithiothreitol (DTT), and 10% glycerol; pH = 7.8). The purity and the identity of proteins were evaluated using Coomassie brilliant blue staining and Western blotting analysis, respectively. Protein samples were divided (20 μL/vial), quickly frozen in liquid nitrogen, and stored at −80 °C. The concentration of purified protein was determined using the Bradford Protein Assay Kit (Beyotime, Beijing, China). All steps of protein purification were performed at 4 °C.

### 2.4. Preparation of Double-Stranded Substrates

The oligonucleotides used to generate double-stranded substrates in this study are presented in Table 1. The 5-carboxyfluorescein (5-FAM)-labeled, tetramethylrhodamine (TAMRA)-labeled, and unlabeled nucleic acids were all synthesized by Tsingke Biotech (Beijing, China) and were purified using high-performance liquid chromatography (HPLC). To generate double-stranded DNA (dsDNA), two complementary single-stranded DNA (ssDNA) were first mixed in the annealing buffer (50 mM Tris and 50 mM NaCl; pH = 8.0); then, the mixture was heated at 95 °C for 5 min, before being cooled down slowly to room temperature. The dsRNA was prepared in the same ways as dsDNA. The detection of the annealing products was conducted using 12% non-denaturing PAGE in Tris-borate-ethylene diamine tetraacetic acid (TBE) buffer (89 mM Tris-borate and 2 mM ethylenediaminetetraacetic acid (EDTA)·Na_2_·2H_2_O)), and the gels were placed into the Typhoon RGB multifunctional laser scanner (Cytiva, Marlborough, MA, USA) for imaging; the gray value of the products was analyzed to determine whether the annealing was complete. Annealing products were diluted into required concentrations with diethylpyrocarbonate (DEPC) water and stored at −20 °C.

### 2.5. Electrophoretic Unwinding Assay

The unwinding activities of NSP13^WT^ and mutants were measured in a reaction containing 30 mM Tris-HCl, 0.1 mg/mL bovine serum albumin (BSA), 1% glycerol, 4 nM substrates, 500 nM trap strand, 3 mM MgCl_2_, 3 mM ATP, 2 mM DTT, and 40 nM purified helicase protein. The reaction was incubated at 37 °C or 15 °C for the indicated time periods and was then terminated by adding the stop buffer containing 50 mM EDTA·Na_2_, 1% sodium dodecyl sulfate (SDS), and 10% glycerol. Here, the trap strand was used to competitively bind the non-fluorescently labeled strand to prevent the annealing of the unwound products, and the sequences of the trap strand were the same as those of the fluorescently labeled single strand. The duplex was denatured at 95 °C for 5 min as a positive control; a reaction without helicase was used as a negative control. Aliquots of each reaction were loaded onto a 12% native polyacrylamide gel in TBE and then electrophoresed at room temperature at 200 V for 2 h. The unwinding products were visualized using a Typhoon RGB multifunctional laser scanner; ImageJ 1.47v software was used to analyze the gray value of the bands to obtain the data of the unwinding reaction. Here, the unwinding results of dsDNA catalyzed by NSP13^WT^ and NSP13^ΔZBD^ were fitted to a single-exponential equation:

*F*(*t*) = *A*× (1 − exp (−*k* × *t*)), (1)

*F*(*t*) is fraction unwound at the reaction time *t*, *A* is the unwinding amplitude, and *k* is the observed rate constant of the burst phase.

### 2.6. ATPase Assay of NSP13^WT^ and Mutants

PDCoV helicase NSP13 is also an ATPase that hydrolyzes ATP to produce energy for the unwinding reaction. In this study, we used the Luminescent Kinase Assay Kit (Beyotime, Shanghai, China) to detect the ATPase activity of NSP13^WT^ and deletion mutants. In total, 5 μM ATP from the reaction buffer (30 mM Tris- HCl, 3 mM MgCl_2_, and 2 mM DTT; pH = 7.5) was added to a 96-well white plate (Labselect, Beijing, China) with deionized water to a total volume of 50 μL; the reaction was initiated by adding NSP13 and was incubated at 25 °C for the specified time. When the reaction time was reached, 50 μL detection reagent was added to the reaction mixture and was incubated at 25 °C for 10 min; the luminescence of each well was measured using a SpectraMax iD5 multifunctional microplate reader (Molecular Devices, San Jose, CA, USA). The detection reagent in this kit contains luciferase, which will be gradually inactivated by repeated freezing and thawing. In order to achieve better results, the detection reagent is dispensed after the first thawing; and the number of freeze-thaw cycles of the dispensed reagent should not be more than three.

### 2.7. Fluorescence Resonance Energy Transfer (FRET)-Based Unwinding Assay

The dsDNA was labeled using two fluorescent dyes and acted as the substrate for the FRET-based unwinding assay; FAM and TAMRA were the donor and acceptor, respectively. For the dsDNA unwinding reaction, 200 nM purified helicase proteins, 40 nM dsDNA, 1 μM trap DNA, 30 mM Tris-HCl, 0.1 mg/mL BSA, 1% glycerol, 3 mM MgCl_2_, and 2 mM DTT were mixed in black 96-well plates. The reaction mixture totaled 100 μL, and the addition of 3 mM ATP was initiated the unwinding reaction. The excitation light was set at 492 nm, the emission light was set at 574 nm, and the photomultiplier tube (PMT) sensitivity was set at high. The total testing time was 30 min, with 1 min intervals. We detected the fluorescent signal of acceptor TAMRA using the Multi-mode Microplate Reader (Molecular Devices, San Jose, CA, USA) to assess the helicase activity of NSP13; the fluorescence value of the receptor decreased when the duplex substrates were unwound.

### 2.8. Sequence Alignment and Structure Prediction

The sequences of different coronaviruses NSP13 were downloaded from the National Center for Biotechnology Information (NCBI) GenBank database, and multiple sequence alignment was carried out using CLUSTALW 2.1 (https://www.genome.jp/tools-bin/clustalw, accessed on 15 October 2024). The illustration of sequence alignment was generated by ESPript 3.0 (https://espript.ibcp.fr/ESPript/cgi-bin/ESPript.cgi, accessed on 15 October 2024). In addition, the structure prediction of NSP13^WT^ and mutants was carried out using the artificial intelligence system AlphaFold2.

## 3. Results

### 3.1. The Sequences of Coronaviruses Helicase NSP13^WT^ Are Highly Conservative

**Figure 1:**
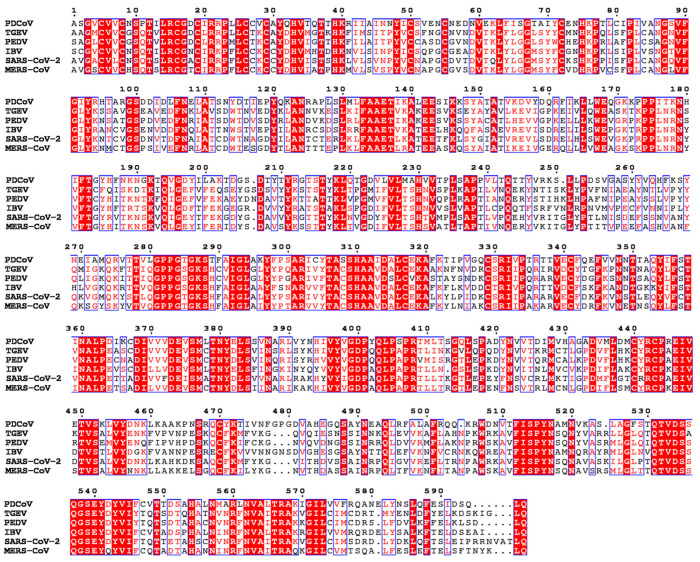
Sequence alignments of coronavirus helicase NSP13^WT^. Alignment was generated by CLUSTALW 2.1 and ESPript 3.0, and the full-length NSP13^WT^ sequences of PDCoV strain CH/JXJGS01/P50 (accession number MH025764.1), TGEV isolate HQ2016 (accession number MT576083.1), PEDV strain AJ1102 (accession number JX188454.1), IBV strain QX (accession number MN548289.1), SARS-CoV-2 isolate Wuhan-Hu-1 (accession number NC_045512.2), and MERS-CoV strain HCoV-EMC (accession number MH454272.1) were derived from the GenBank database.

To identify the sequence conservation of the helicase NSP13 of coronaviruses, the full-length NSP13^WT^ sequences of transmissible gastroenteritis virus (TGEV) isolate HQ2016, PEDV strain AJ1102, infectious bronchitis virus (IBV) strain QX, SARS-CoV-2 isolate Wuhan-Hu-1, and MERS-CoV strain HCoV-EMC were aligned (Figure 1). Overall, the protein alignment revealed that the sequence identity of the helicase NSP13^WT^ among these coronaviruses was 34.82%, and the sequence similarity was 82.18%. Moreover, the sequence alignment of NSP13 across six PDCoV strains demonstrated complete conservation (100% identity) (Figure S1). These results indicate that the helicase NSP13 among different coronaviruses is highly conserved, and it can be an ideal target for the development of broad-spectrum anti-coronavirus drugs.

### 3.2. The Structures of Helicase NSP13^WT^ and Various Mutants

**Figure 2:**
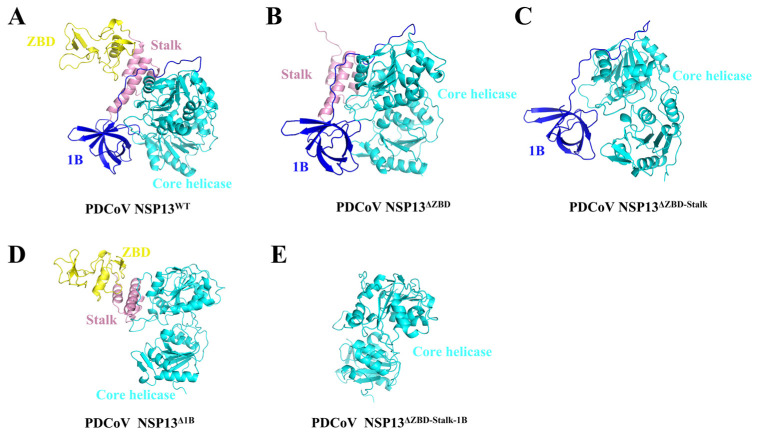
Overall structure of full-length PDCoV NSP13^WT^ and deletion mutants. (**A**) Ribbon structure of PDCoV helicase NSP13^WT^. (**B**) Ribbon structure of NSP13^ΔZBD^ with the deletion of the ZBD. (**C**) Ribbon structure of NSP13^ΔZBD-Stalk^ with the deletion of both the ZBD and 1B domain. (**D**) Ribbon structure of NSP13^Δ1B^ with the deletion of the 1B domain. (**E**) Ribbon structure of NSP13^ΔZBD-Stalk-1B^ with the deletion of the three domains at the amino terminal. Helicase domains in figures were presented in different colors—ZBD (yellow), Stalk domain (pink), 1B domain (blue), and core helicase domain (cyan).

Before conducting formal experiments to identify the role of different domains in the activities of PDCoV helicase NSP13^WT^, the three-dimensional (3D) structures of full-length NSP13^WT^ (Figure 2A) and various deletion mutants were predicted using the artificial intelligence system AlphaFold2. Here, the mutants contained NSP13^ΔZBD^ (Figure 2B), NSP13^ΔZBD-Stalk^ (Figure 2C), NSP13^Δ1B^ (Figure 2D), and NSP13^ΔZBD-Stalk-1B^ (Figure 2E). As shown in Figure 2, the full-length NSP13^WT^ consisted of a ZBD, a Stalk domain, a 1B domain, and a core helicase domain; the ZBD and 1B domain were connected through the Stalk domain. The Stalk, 1B, and core helicase domains worked together to form a relatively “close” conformation, and the deletion of either domain may lead to a conformation alteration that could possibly affect NSP13^WT^ activities.

### 3.3. Determination of the Unwinding and ATPase Activities of PDCoV NSP13^WT^

**Figure 3:**
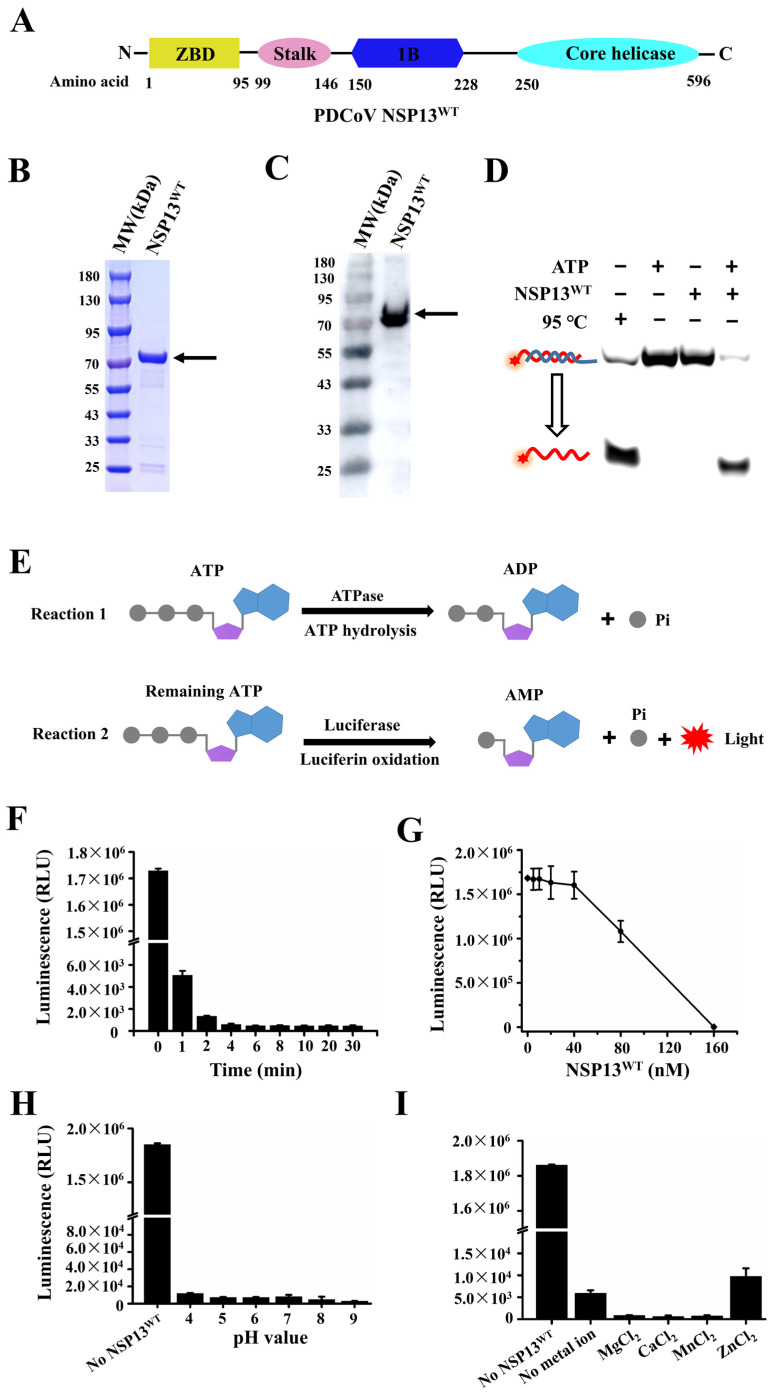
PDCoV NSP13^WT^ had the ability to unwind the duplex and hydrolyze ATP. (**A**) Schematic diagram of the domain organization of NSP13^WT^. (**B**) SDS-PAGE of purified products. (**C**) Western blotting of helicase NSP13^WT^. (**D**) Detection of the unwinding activity of NSP13^WT^. (**E**) Schematic diagram of the determination of the ATPase activity of NSP13^WT^. (**F**) Measurement of the ATPase activity at different time points. (**G**) ATPase activity was measured at different concentrations of NSP13^WT^. (**H**) Identifying the effect of solution pH on the ATPase activity. (**I**) Identifying the effect of metal icons on the ATPase activity. The black arrows and red stars indicate the target protein NSP13^WT^ and 5′-FAM, respectively. The error bars represent the standard deviation.

The helicase NSP13^WT^ of PDCoV comprised 596 amino acids; a schematic diagram of NSP13^WT^ is shown in Figure 3A. In this study, recombinant NSP13^WT^ of PDCoV containing a His-tag was expressed in *Escherichia coli* and purified using Nickel-Nitrilotriacetic Acid (Ni-NTA) chromatography. The purified products were identified using sodium dodecyl sulfate-polyacrylamide gel electrophoresis (SDS-PAGE) and Western blotting, and the positions of bands in the gels were generally consistent with the predicted molecular weight (MW) (70 kDa) of the target protein NSP13^WT^ (Figure 3B,C). Subsequently, the unwinding activity of NSP13 was tested using partial duplex DNA as a model and control substrate. Since the advantages of dsDNA include its simple manipulation, low price, and non-degradability, we focused on the function of PDCoV NSP13^WT^ to unwind dsDNA substrates in the subsequent experiments. The results showed that NSP13^WT^ unwound dsDNA in an ATP-dependent manner, and no visible unwinding of dsDNA occurred in the absence of ATP (Figure 3D).

ATPase activity is one of the key functions of helicases, and most helicases use the energy from ATP hydrolysis to unwind the helixes of substrates. Here, the ATPase activity of NSP13^WT^ was measured using ATP hydrolysis combined with luciferin oxidation reactions; the chemiluminescence value of the assay was detected to determine the ATPase activity (Figure 3E). The results showed that PDCoV helicase NSP13^WT^ rapidly hydrolyzed ATP in a time-dependent manner, and the amount of ATP remaining in the reaction mixture immediately decreased, reaching its lowest level after 4 min (Figure 3F). The ATPase activities of different concentrations of NSP13^WT^ (0, 5, 10, 20, 40, 80, and 160 nM) were also tested, and the amount of ATP decreased slowly in the low concentration range of NSP13^WT^; the ATP was sharply hydrolyzed as the NSP13^WT^ concentrations increased to more than 40 nM (Figure 3G). Moreover, the reaction without NSP13^WT^ was used as an experimental control, and we determined the amount of ATP in solutions of different pH (4, 5, 6, 7, 8, and 9); the results showed that the solution pH had almost no effect on the ATPase activity of NSP13^WT^ (Figure 3H). Divalent metal ions are important cofactors for enzymes, and the preference for ions may vary among enzymes. To evaluate the metal requirements for the ATPase activity, NSP13^WT^ was incubated with 5 μM ATP in the presence of 3 mM MgCl_2_, CaCl_2_, MnCl_2_, and ZnCl_2_. The luminescence measurements showed that NSP13^WT^ could hydrolyze ATP independently of metal ions, but the addition of metal ions (MgCl_2_, CaCl_2_, and MnCl_2_) facilitated the hydrolysis of ATP, while ZnCl_2_ exhibited an inhibitory effect on ATPase activity (Figure 3I). In contrast, we also found that PDCoV NSP13^WT^ required Mg^2+^ to unwind duplex substrates (Figure S2), which was consistent with the SARS-CoV-2 helicase NSP13 [16]. The data indicated that divalent metal ions were indispensable for the unwinding activity of PDCoV NSP13^WT^, but not the ATPase activity.

### 3.4. The Deletion of the ZBD Impaired the Unwinding Activity of PDCoV Helicase NSP13^WT^

**Figure 4:**
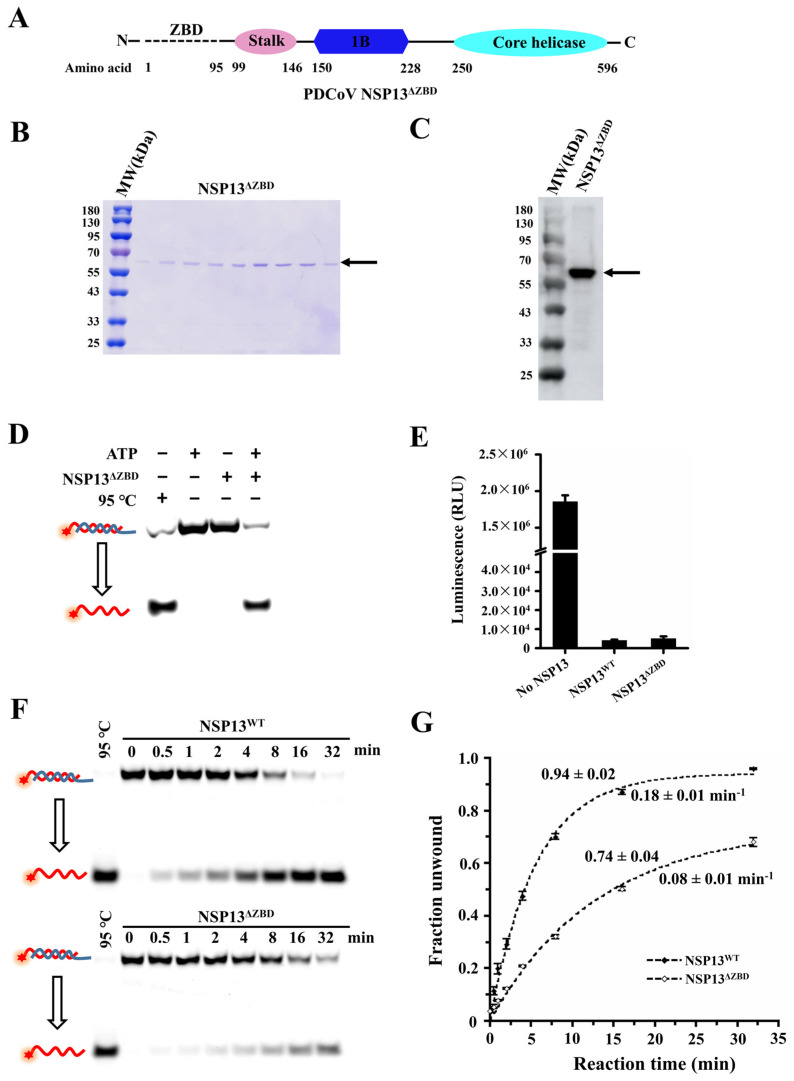
Determination of the unwinding and ATPase activities of the mutant NSP13^ΔZBD^. (**A**) Schematic diagram of the domain organization of mutant NSP13^ΔZBD^. Dotted lines represent the deleted region. (**B**) Purified products were detected using SDS-PAGE. (**C**) Target protein was verified using Western blotting. (**D**) The substrate dsDNA was unwound by NSP13^ΔZBD^ in the presence of ATP. (**E**) ATP was hydrolyzed by NSP13^ΔZBD^. (**F**) The dsDNA was unwound by NSP13^WT^ and NSP13^ΔZBD^, and the reaction products were resolved on a 12% native-PAGE gel. (**G**) Fitting curves of unwinding fraction and reaction time. The black arrows point to the target protein NSP13, while the red stars indicate 5′-FAM. The error bars represent the standard deviation.

The ZBD of coronaviruses helicase NSP13^WT^ is located at the amino terminus and coordinates three structural zinc ions [26]. However, whether the ZBD is critical for the activities of PDCoV NSP13^WT^ remains unknown. To answer this question, we expressed the mutant NSP13^ΔZBD^ with the ZBD deleted (Figure 4A) and explored the activities of NSP13^ΔZBD^. The purified products were detected on the SDS-PAGE gel and the position of the bands was essentially the same as the expected molecular weight of NSP13^ΔZBD^ (60 kDa) (Figure 4B). Then, the mutant was verified using Western blotting (Figure 4C).

To determine whether the deletion of the ZBD affects the unwinding activity of NSP13, dsDNA and NSP13^ΔZBD^ were incubated in the unwinding buffer in the presence of ATP or in the absence of ATP. The results showed that NSP13^ΔZBD^ was able to efficiently unwind dsDNA in an ATP-dependent manner (Figure 4D). Furthermore, the residual ATP in the reaction buffer was detected by measuring chemiluminescence values to determine the ATPase activity of NSP13^ΔZBD^. Compared to the controls with NSP13 or without NSP13, we found that the mutant NSP13^ΔZBD^ still had a good ability to hydrolyze ATP (Figure 4E). These data indicated that the ZBD was not essential for the unwinding and ATPase activities of PDCoV helicase NSP13.

However, our study only qualitatively analyzed the activity of NSP13^ΔZBD^, not quantitatively. Therefore, we performed titration experiments using dsDNA catalyzed by NSP13^WT^ and NSP13^ΔZBD^ in order to determine the effect of ZBD deletion on the unwinding activity. Notably, due to the unwinding rate being too fast for data acquisition at 37 °C, a temperature of 15 °C was used for the titration experiments. The results showed that NSP13^WT^ or NSP13^ΔZBD^ unwound dsDNA in a time-dependent manner (Figure 4F). The unwinding amplitude and rate of dsDNA by NSP13^WT^ were *A*_1_ = 0.94 ± 0.02 and *k*_1_ = 0.18 ± 0.01 min^−1^, respectively. Compared with NSP13^WT^, the unwinding amplitude and rate of dsDNA catalyzed by mutant NSP13^ΔZBD^ were significantly reduced to *A*_2_ = 0.74 ± 0.04 and *k*_1_ = 0.08 ± 0.01 min^−1^ (Figure 4G). Moreover, NSP13^ΔZBD^ exhibited a weaker activity when unwinding dsRNA compared to NSP13^WT^ (Figure S3). Based on these results, we found that the deletion of the ZBD impaired the unwinding activity of PDCoV helicase NSP13^WT^.

### 3.5. The Stalk and 1B Domains Are Essential for Unwinding Activity

The Stalk domain of SARS-CoV-2 NSP13^WT^ provides a rigid connection between the ZBD and the helicase domain, and the linker insertion between the stalk and helicase domains affects RNA binding activity and NSP13 stability [27]. Nevertheless, the function and mechanism of the stalk domain of PDCoV helicase NSP13^WT^ remain unclear.

**Figure 5:**
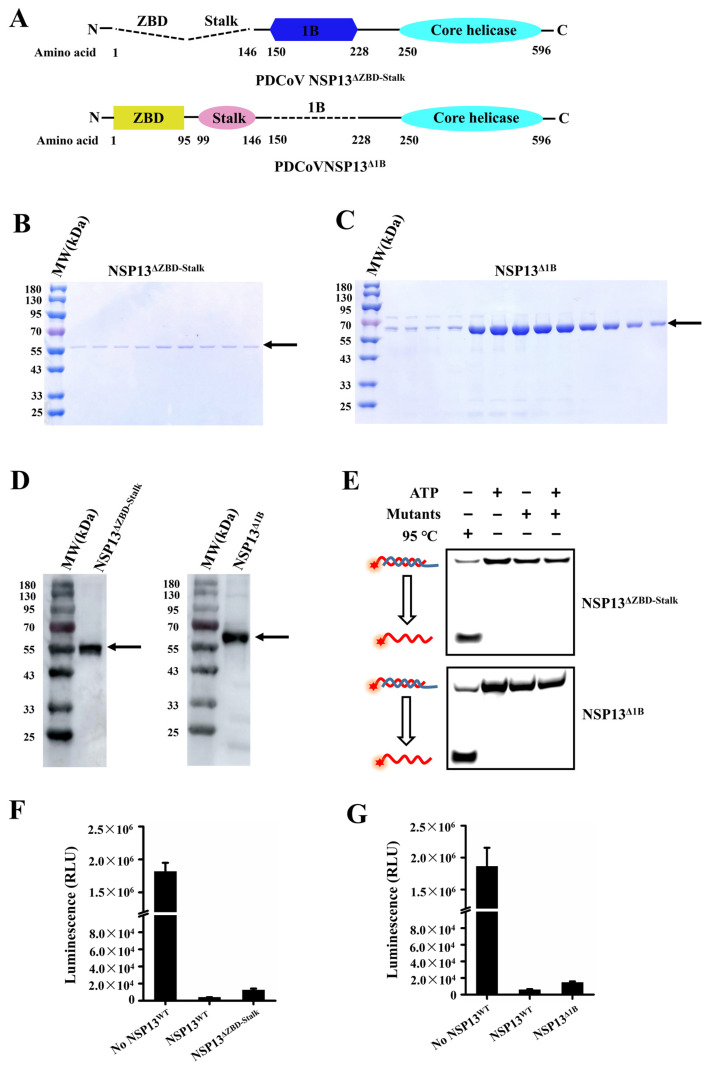
Demonstrating the role of the Stalk and 1B domains in the activities of PDCoV NSP13^WT^. (**A**) Schematic diagram of the domain organization of mutant NSP13^ΔZBD-Stalk^ and NSP13^Δ1B^. Dotted lines represent the deleted region. (**B**) SDS-PAGE analysis of the purified NSP13^ΔZBD-Stalk^; the gel was stained with Coomassie Blue. (**C**) Purified NSP13^Δ1B^ was detected using SDS-PAGE. (**D**) NSP13^ΔZBD-Stalk^ (left) and NSP13^Δ1B^ (right) were confirmed using Western blotting. (**E**) The unwinding activities of NSP13^ΔZBD-Stalk^ (top) and NSP13^Δ1B^ (bottom) were tested. (**F**) Determination of the ATPase activity of the mutant NSP13^ΔZBD-Stalk^. (**G**) The ATPase activity of the mutant NSP13^Δ1B^ was measured. The arrows in the figures point to the target proteins, while the red stars indicate 5′-FAM. The error bars represent the standard deviation.

We expressed a recombinant helicase mutant NSP13^ΔZBD-Stalk^ with a His-tag in *Escherichia coli* that lacked both the ZBD and Stalk domain (Figure 5A). The truncated NSP13^ΔZBD-Stalk^ was analyzed using electrophoresis on SDS-PAGE (Figure 5B) and Western blotting with anti-His antibody (Figure 5D, left), and the bands represented the purified NSP13^ΔZBD-Stalk^ with an expected mass of 54kDa. To confirm the role of the Stalk domain in the unwinding and ATPase function of NSP13^WT^, DNA unwinding and ATP hydrolyzing assays were performed. The results showed that no dsDNA was unwound by NSP13^ΔZBD-Stalk^ (Figure 5E, top), but the ATP hydrolysis activity was not affected (Figure 5F); this verified that the deletion of the Stalk domain abolished the unwinding activity of PDCoV NSP13^WT^.

Subsequently, the effect of the deletion of the 1B domain on NSP13^WT^ activities was explored. The purified mutant NSP13^Δ1B^ (62kDa) was abundantly expressed and purified, and the purified NSP13^Δ1B^ was analyzed using SDS-PAGE (Figure 5C) and Western blotting (Figure 5D, right). Moreover, the unwinding and ATPase activities of NSP13^Δ1B^ were also tested, and we found that NSP13^Δ1B^ was unable to unwind dsDNA in the presence of ATP (Figure 5E, bottom), but ATP was hydrolyzed by NSP13^Δ1B^ (Figure 5G). The results suggested that the 1B domain was critical for the unwinding activity of PDCoV helicase NSP13^WT^.

### 3.6. The Independent Core Helicase Domain Retains Only the Ability to Hydrolyze ATP

The carboxyl-terminal core helicase domain of NSP13^WT^ consisted of Rec1A and Rec2A, which are the shared domains of SF1 family helicases. Rec1A is composed of I, Ia, Ib, II, and III conserved motifs, while Rec2A contains motifs IV, V, and VI. The core helicase domain is important for NSP13^WT^ activities, and there are multiple key active sites on the core helicase domain; however, it remains unclear whether the core helicase domain has abilities to unwind dsDNA and hydrolyze ATP alone.

**Figure 6:**
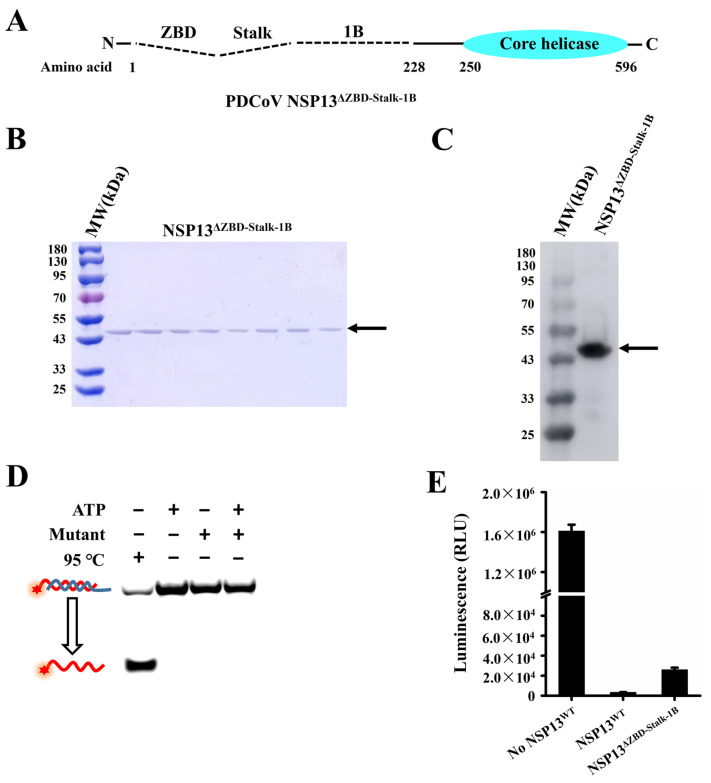
The independent core helicase domain had lost the ability to unwind double-stranded substrates. (**A**) Schematic representation of the mutant NSP13^ΔZBD-Stalk-1B^ with the independent core helicase domain. Dotted lines represent the deleted region. (**B**) SDS-PAGE analysis of the purified NSP13^ΔZBD-Stalk-1B^ protein. (**C**) Purified NSP13^ΔZBD-Stalk-1B^ was confirmed using Western blotting. (**D**) Determination of the unwinding activity of NSP13^ΔZBD-Stalk-1B^. (**E**) The ATPase activity of NSP13^ΔZBD-Stalk-1B^ was detected. The arrows in the figures point to the target protein NSP13^ΔZBD-Stalk-1B^, while the red stars indicate 5′-FAM. The error bars represent the standard deviation.

Therefore, we deleted the ZBD, as well as the Stalk and 1B domains, of PDCoV helicase NSP13^WT^, and the mutant NSP13^ΔZBD-Stalk-1B^ containing only a core helicase domain was expressed (Figure 6A). The purified protein NSP13^ΔZBD-Stalk-1B^ (45kDa) was confirmed using SDS-PAGE and Western blotting (Figure 6B,C). In addition, the unwinding and ATPase assays were used to test the mutant helicase activities. The results showed that no dsDNA was unwound by NSP13^ΔZBD-Stalk-1B^ (Figure 6D), but the amount of ATP was dramatically decreased (Figure 6E). These data revealed that the independent core helicase domain with the deletion of the three carboxyl terminal domains (i.e., the mutant NSP13^ΔZBD-Stalk-1B^) abolished the unwinding activity of NSP13^WT^; however, ATPase activity was almost unaffected.

### 3.7. The Unwinding Activities of NSP13^WT^ and Mutants Were Tested Using FRET-Based Assays

**Figure 7:**
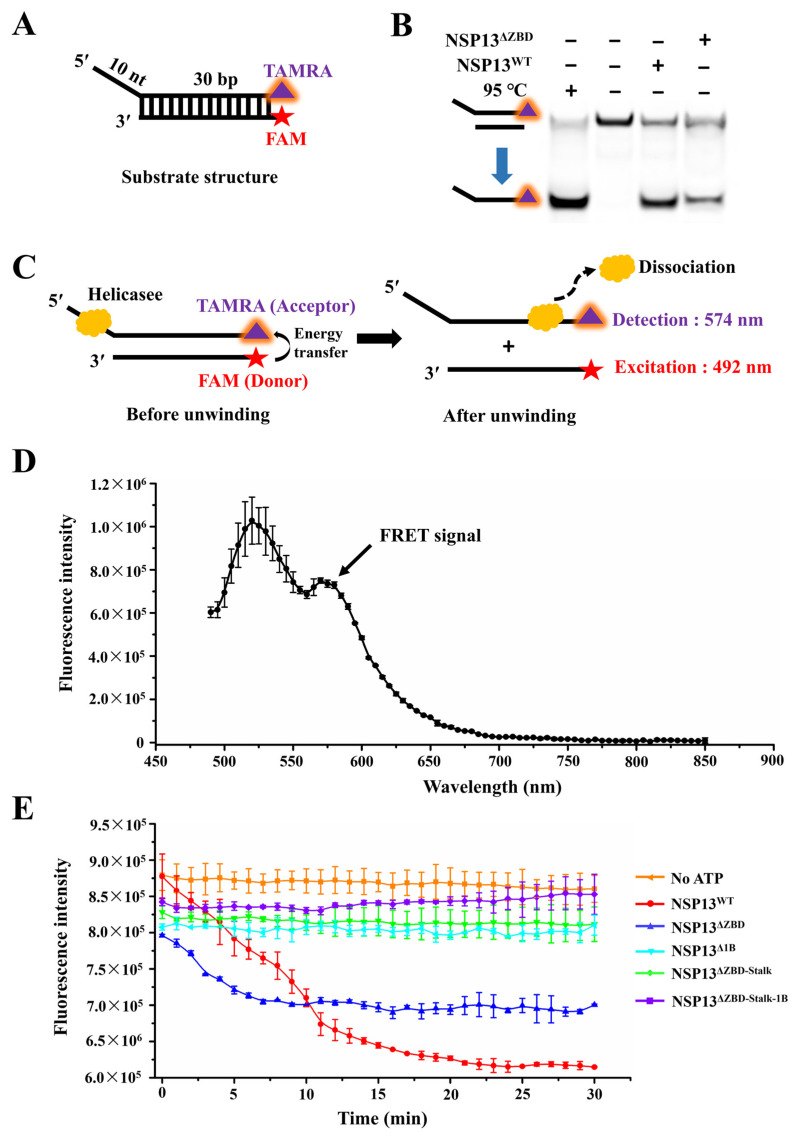
Detection of the unwinding activity of NSP13^WT^ and mutants using the FRET assay. (**A**) The structure of the substrate used in the FRET assay. The purple and cyan triangles represent TAMRA and FAM, respectively. (**B**) The unwinding products were separated on an SDS-PAGE gel and visualized through the laser excitation of TAMRA. (**C**) Schematic of the FRET-based dsDNA unwinding assay. The yellow graph indicates the helicase. (**D**) Fluorescence spectrum of FAM and TAMRA. The arrow indicates that a FRET was generated. (**E**) The unwinding reaction of NSP13^WT^ and different mutants in the presence of ATP, and the reaction without ATP as the negative control. The red pentagrams denote FAM, and the yellow triangles indicate TAMRA. The error bars represent the standard deviation.

To further confirm the effect of the deletions of different domains on the unwinding activity of PDCoV helicase NSP13, we also observed the real-time separation of dsDNA using FRET-based assays. Here, a 30 bp dsDNA with a 10 nt 5′-overhang was designed, and the fluorescent dyes FAM and TAMRA were simultaneously labeled on the same side of the dsDNA (Figure 7A). To ensure that the subsequent FRET-based unwinding experiments were feasible, we first demonstrated that both NSP13^WT^ and NSP13^ΔZBD^ were able to unwind this dsDNA, and the latter appeared to have a lower unwinding efficiency (Figure 7B).

Furthermore, the dsDNA unwinding activities of NSP13^WT^ and various mutants were also measured based on the FRET from the FAM (donor) to the TAMRA (acceptor). More specifically, our experimental setup was devised in such a way that the fluorescence from TAMRA was generated when the two DNA strands were base-paired, but a reduced fluorescence from TAMRA was detectable due to the disappearing FRET when the dsDNA was unwound by helicases (Figure 7C). Based on this principle, the unwinding activities were measured via the detection of the emitting fluorescent intensity at a wavelength of 574 nm. Before the unwinding experiments, the FRET signal of substrate dsDNA was first detected using ensemble FRET. FAM was excited at a wavelength of 492 nm and the fluorescence emission was measured from 450 to 850 nm; the emission step was 5 nm. Here, we observed the FRET signal, and a distinct peak at ~575 nm proved that energy transfer from donor to acceptor occurred when exciting the donor FAM (Figure 7D). These data indicated that the dsDNA could be used for the FRET-based unwinding assay. Then, we tested the unwinding activities of NSP13^WT^ and mutants, and the results showed a significant decrease in the fluorescent intensity in the NSP13^WT^-catalyzed reaction; the fluorescent intensity was also reduced in the presence of NSP13^ΔZBD^. However, the fluorescent intensity in the unwinding reactions containing the other mutants (NSP13^ΔZBD-Stalk^, NSP13^ΔZBD-Stalk-1B^, and NSP13^Δ1B^) remained almost constant. Notably, differences in the initial fluorescence intensity of the reactions might be related to the ability of the enzyme to bind the nucleic acids. Overall, these results indicated that deletions of the Stalk and 1B domains were lethal to the unwinding activity of the PDCoV helicase NSP13, and the deletion of the ZBD reduced this ability (Figure 7E).

## 4. Discussion

PDCoV is a member of the animal coronaviruses family that may have zoonotic potential, and the development of effective antiviral drugs has become one of the important means to treat virus infections [28]. Some NSPs encoded by coronaviruses have become ideal targets, including the polymerase NSP12, the protease NSP5, and the helicase NSP13 [29,30]. NSP13 has multiple enzymatic functions and is considered as a key enzyme for viral replication, and the unwinding activity of NSP13 is thought to be critical for clearing the secondary structure of nucleic acids during replication and transcription. Coronavirus replication can be disrupted by inhibiting NSP13 functions, and NSP13 inhibition can be achieved through breaking the NSP13’s abilities to bind and hydrolyze ATP, interfering with the binding of nucleic acids to helicase, blocking helicases translocation and unwinding, etc. [31,32]. The domains of NSP13 contain multiple active sites that affect the normal function of NSP13, but the specific role of each domain remains unclear.

**Figure 8:**
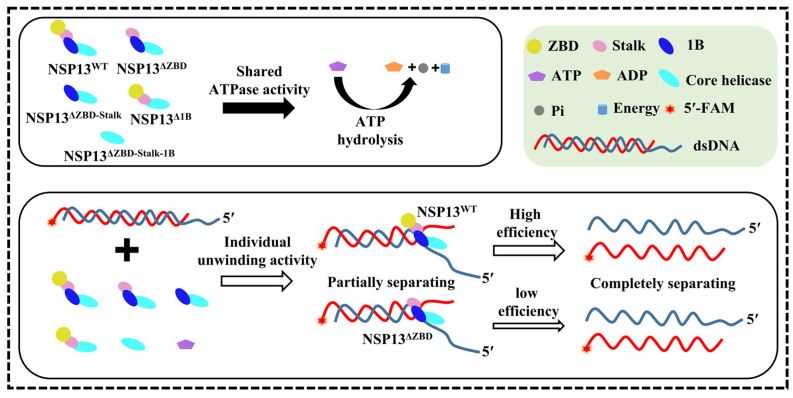
Model showing NSP13 and mutants hydrolyzing ATP and unwinding dsDNA. PDCoV helicase NSP13^WT^ and four deletion mutants are all able to hydrolyze ATP, but only NSP13^WT^ and NSP13^ΔZBD^ exhibits the ability to unwind dsDNA. Compared with NSP13^WT^, the unwinding efficiency of NSP13^ΔZBD^ is reduced by the deletion of the ZBD. The solid arrow points to the reaction of ATP hydrolysis (**top**), and the hollow arrows point to the unwinding reaction (**bottom**). The thickness of the hollow arrows indicates the level of unwinding efficiency.

In this paper, we expressed and purified the full-length PDCoV helicase NSP13^WT^, as well as various mutants with domain deletions, and measured the unwinding and ATPase activities of NSP13^WT^ and these mutants. The results showed that NSP13^WT^ unwound double-stranded nucleic acids in an ATP-dependent manner, but NTPase activity could be measured without nucleic acid, indicating that the unwinding and NTPase activities of NSP13^WT^ were uncoupled, which was consistent with a study involving SARS-CoV-2 helicase [33]. The model presented in Figure 8 demonstrated that PDCoV NSP13^WT^ and its truncation variants were all capable of hydrolyzing ATP, but only NSP13^ΔZBD^ exhibited the ability to unwind duplex nucleic acids, albeit with a lower efficiency compared to NSP13^WT^.

The amino-terminal ZBD is an accessory domain that is unique to the viral order *Nidovirales* and has been shown to coordinate three Zn^2+^ ions by conserved cysteine and histidine residues in three zinc fingers [26,34,35]. Proposed ZBD inhibitors impaired the unwinding and ATPase activities of helicase by disrupting the bond between Zn^2+^ ions and the coordinating residues in the zinc fingers [19]. Mutations in the ZBD of the equine arteritis virus (EAV) impaired helicase enzymatic activity and RNA production during viral replication [36]. Here, we found that NSP13^ΔZBD^ with the deletion of the ZBD still had the ability to hydrolyze ATP and unwind duplex nucleic acids, but the unwinding efficiency of NSP13^ΔZBD^ was lower than that of full-length NSP13^WT^ (Figure 8). These results revealed that the deletion of the ZBD merely weakened the activities of PDCoV helicase NSP13^WT^, which was similar to the conclusions drawn from previous studies.

The Stalk domain was shown to be critical to virus survival, and an arginine-to-proline mutation at position 132 in the Stalk domain of IBV helicase NSP13 caused the virus to lose infectivity and stop proliferating [37]. In addition, the 1B domain may coordinate the helicase with template nucleic acid, and mutations in the 1B domain are reported to have impaired enzymatic activity [38]. For instance, the T216A mutation of the 1B domain impaired the unwinding activity of SARS-CoV-2 NSP13 [39]. In our current study, the deletion of either the Stalk or the 1B domain resulted in the complete loss of the unwinding activity of PDCoV NSP13^WT^, but mutants still retained their ATPase activity (Figure 8). In contrast, the deletion of the Salk domain of SARS-CoV-2 NSP13 impaired the ATPase activity and eliminated the unwinding activity [27]. These results indicated that the Stalk and 1B domains were critical for NSP13 activities. Nevertheless, it was not clear whether the deletion of these domains specially affected helicase–substrate binding, helicase translocation, or the breaking of hydrogen bonds.

Together, Rec1A and Rec2A form the core helicase domain of coronavirus helicase, and the motifs Ia, IV, and V of the core helicase domain were critical for the ATP-dependent inchworm translocation mechanism of helicase [40]. Mutations in key amino acid sites of Rec1A or Rec2A disrupted the binding of the helicase to the nucleic acids and impaired the unwinding and ATPase activities of NSP13^WT^ [26,41]. Here, we deleted all three amino-terminal domains of PDCoV NSP13^WT^ and found that the helicase mutant lost its unwinding activity, but not its ATPase activity (Figure 8). These data indicated that the motifs on the core helicase domain were sufficient for the binding and hydrolysis of ATP, which also explained why ATPase activity was not abolished by either the deletion of the amino-terminal domains individually or by the simultaneous deletion of multiple domains. Moreover, this paper also verified that lysine at position 287 was required for the activities of PDCoV NSP13 (Figure S4), which was consistent with studies relating to PEDV and MERS-CoV helicases [14,17]. Taken together, the biochemical analysis of NSP13 and various mutants had advanced the clarification of the function of the domains, as well as the understanding of the role of the helicase in viral replication, providing a theoretical basis for antiviral development.

## 5. Conclusions

In this work, to experimentally explore the role of different domains in the activities of PDCoV helicase NSP13, wild-type NSP13^WT^ and multiple deletion mutants were expressed in *Escherichia coli* with hexa-histidine tags at the N-terminus and were purified using affinity chromatography. The function of each domain was determined by comparing the differences in the activities of NSP13^WT^ and the mutants. We found that the unwinding efficiency of NSP13^ΔZBD^ decreased, and NSP13^ΔZBD-Stalk^, NSP13^ΔZBD-Stalk-1B^, and NSP13^Δ1B^ all lost their unwinding activity. In contrast, all mutants with various domain deletions still possessed ATPase activity. The results indicated that the deletion of the ZBD weakened the unwinding activity of PDCoV helicase NSP13, while the deletion of the Stalk or 1B domain caused NSP13 to completely lose its ability to unwind double-stranded nucleic acids. Furthermore, the core helicase domain alone was not capable of unwinding duplex substrates. In addition, the ZBD, as well as the Stalk and 1B domains, were not required for ATP hydrolysis catalyzed by NSP13, and the ATPase activity might mainly rely on the core helicase domain.

## Figures and Tables

**Table 1:** Oligonucleotides used in this study.

Names	Sequences	Nts
DNA1	5′-★-GCGACGTCACGTGCA-3′	15
DNA2	5′-CAGCTAGACCTGCACGTGACGTCGC-3′	25
RNA1	5′-★-GCGUCGUAUCGAUCU-3′	15
RNA2	5′-CAGCUAGACCAGAUCGAUACGACGC-3′	25
DNA3	5′-★-ACCTGGATTGGTGTCGGTAGAGAACTAGCG -3′	30
DNA4	5′-TCCCTAGCTTCGCTAGTTCTCTACCGACACCAATCCAGGT-▲-3′	40
Trap DNA1	5′-GCGACGTCACGTGCA-3′	15
Trap DNA2	5′-ACCTGGATTGGTGTCGGTAGAGAACTAGCG-3′	30
Trap RNA	5′-GCGUCGUAUCGAUCU-3′	15

Note: the pentagrams denote FAM, and the triangle indicates TAMRA.

## Data Availability

All data generated during this study are included in this published article and its Supplementary Information files.

## Supplementary Materials

The following supporting information can be downloaded at: https://www.mdpi.com/article/10.3390/ani15060865/s1. Figure S1: Alignment of NSP13 amino acid sequences among six PDCoV strains; Figure S2: A magnesium ion is essential for the unwinding activity of PDCoV NSP13; Figure S3: Both NSP13 and mutant NSP13^ΔZBD^ were able to unwind dsRNA; Figure S4: Detection of the helicase activity and ATPase activity of mutant NSP13^K287A^.

## Abbreviations

The following abbreviations are used in this paper:

BSA	Bovine serum albumin
3CLpro	3-chymotrypsin-like protease
DEPC	Diethylpyrocarbonate
dsDNA	Double-stranded DNA
DTT	Dithiothreitol
EAV	Equine arteritis virus
EDTA	Ethylenediaminetetraacetic acid
FAM	Carboxyfluorescein
FRET	Fluorescence resonance energy transfer
HPLC	High-performance liquid chromatography
IBV	Infectious bronchitis virus
LB	Luria–Bertani
MERS-CoV	Middle East respiratory syndrome coronavirus
NCBI	National Center for Biotechnology Information
Ni-NTA	Nickel-Nitrilotriacetic acid
NSP13	Nonstructural protein 13
NTP	Nucleotide trisphosphate
PAGE	Polyacrylamide gel electrophoresis
PBS	Phosphate-buffer saline
PDCoV	Pocine deltacoronavirus
PEDV	Porcine epidemic diarrhea virus
PLpro	Papain-like protease
PMT	Photomultiplier tube
Rec1A	Recombination-like domain 1A
SARS-CoV	Severe acute respiratory syndrome coronavirus
SDS	Sodium dodecyl sulfate
ssDNA	Single-stranded DNA
TAMRA	Tetramethylrhodamine
TBE	Tris-borate-ethylene diamine tetraacetie acid
TGEV	Transmissible gastroenteritis virus
Upf1	Upstream frameshift protein 1
ZBD	Zinc-binding domain
