# Supplementary material for: The Stalk and 1B Domains Are Required for Porcine Deltacoronavirus Helicase NSP13 to Separate the Double-Stranded Nucleic Acids, and the Deletion of the ZBD Impairs This Activity
Source: Animals (Basel). 2025 Mar 18;15(6):865. doi: 10.3390/ani15060865 (PMC11939599; doi:10.3390/ani15060865)
Supplement: Supplementary file 1 [file animals-15-00865-s001.zip › animals-3478884-supplementary.pdf]

# Supplementary Materials

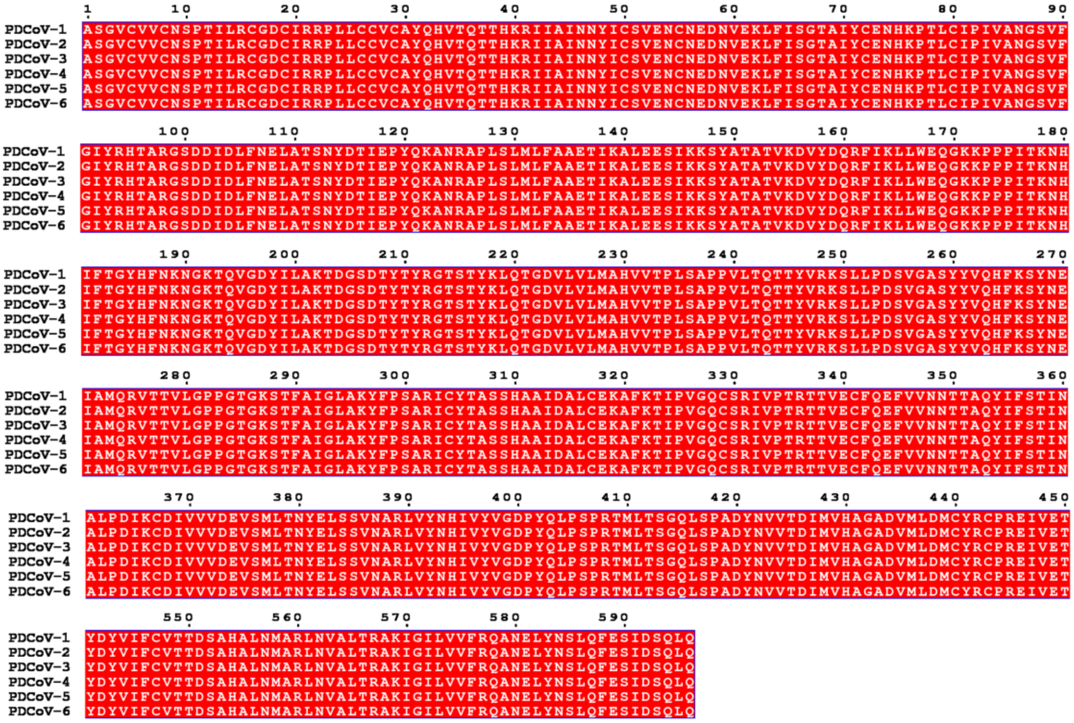

**Figure S1** Alignment of NSP13 amino acid sequences among six PDCoV strains. The PDCoV strains were derived from different regions, and NSP13 sequences of different strains were downloaded from GenBank database, contain the strain CH/JXJGS01/P50 (accession number MH025764.1) in China, strain CH/Jiangsu/2014 (accession number KY513725.1) in China, isolate CHN-HN-1601 (accession number MG832584.1) in China, isolate KNU16-07 (accession number KY364365.1) in South Korea, strain OH-FD22 P7 (accession number MZ291567.1) in United States, and isolate P19_16_VN_0416 (accession number MH118332.1) in Vietnam. The alignment results showed that the NSP13 sequences of different PDCoV strains were identical.

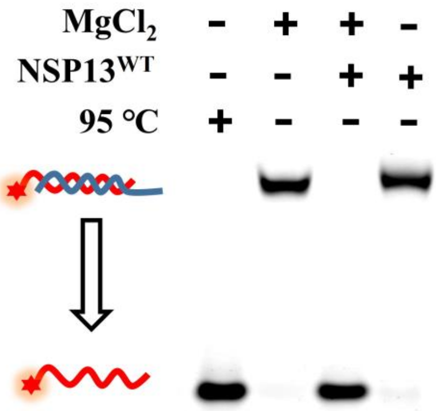


**Figure S2** Magnesium ion is essential for the unwinding activity of PDCoV NSP13. 40 nM purified NSP13 was incubated with 4 nM dsDNA in the unwinding buffer at 37 °C for 30 min. The dsDNA was denatured at 95 °C as a

positive control, and the unwinding reaction without NSP13 as a negative control. The results showed that dsDNA was unwound by NSP13 in the presence of $Mg^{2+}$.

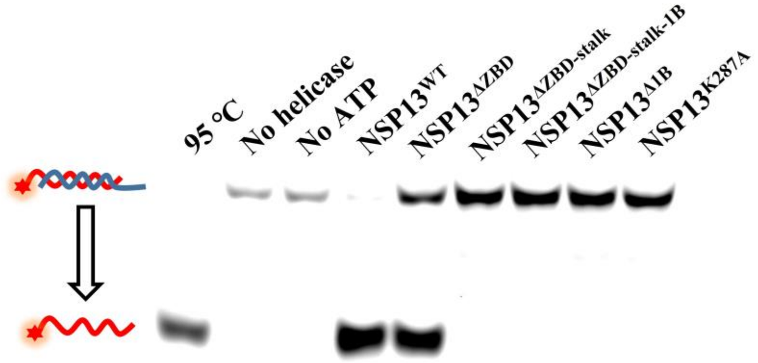


**Figure S3** Both NSP13 and mutant $NSP13^{\Delta ZBD}$ were able to unwind dsRNA. 40 nM purified NSP13 or different mutants were incubated with 4 nM 5′-10nt-15bp dsRNA in the unwinding buffer at 37 °C for 30 min. The results showed that dsRNA was unwound by NSP13 or $NSP13^{\Delta ZBD}$, but not other mutants.

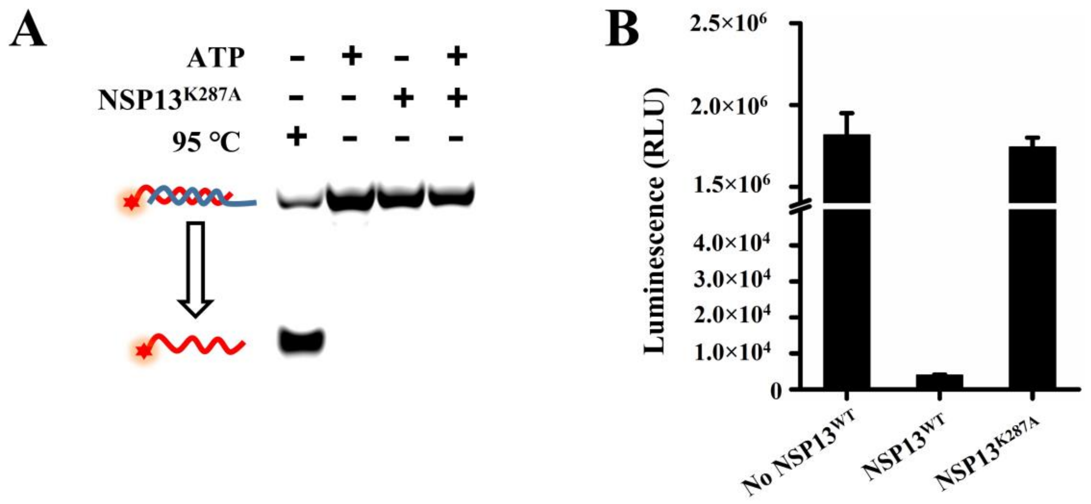


**Figure S4.** Detection of the helicase activity and ATPase activity of mutant $NSP13^{K287A}$. **A.** Duplex substrate was not unwound by $NSP13^{K287A}$. Substrate was treated at 95 °C as a positive control, and unwinding reaction without helicase as a negative control. The asterisk in the figure denotes the FAM. **B.** K287A mutation of NSP13 almost completely abolished the ATPase activity.
